# Knowledge, attitudes, and practices toward Patient Safety among nurses in health centers

**DOI:** 10.1186/s12912-024-01831-1

**Published:** 2024-03-14

**Authors:** Ahmad Ayyad, Nesrin Abu Baker, Islam Oweidat, Khalid Al-Mugheed, Samira Ahmed Alsenany, Sally Mohammed Farghaly Abdelaliem

**Affiliations:** 1 School of Nursing, Community and Mental Health Department, Jordan University of Science and Technology, 22110 Irbid, P. O. Box 3030, Jordan; 2 Community and Mental Health Nursing Department, Faculty of Nursing, Zarqa- Jordan, Zarqa, Jordan; 3 College of Nursing,Riyadh Elm University, Riyadh, Saudi Arabia; 4 Department of Community Health Nursing, College of Nursing, Princess Nourah bint Abdulrahman University, 11671 Riyadh, P. O. Box 84428, Saudi Arabia; 5 Department of Nursing Management and Education, College of Nursing, Princess Nourah bint Abdulrahman University, 11671 Riyadh, P. O. Box 84428, Saudi Arabia

**Keywords:** Patient safety, Knowledge, Attitudes, Practices, Jordan, Nurse

## Abstract

**Objective:**

To assess knowledge, attitudes, and practices (KAPs) toward patient safety among nurses working at primary and comprehensive health care centers in Jordan; to identify factors that predict KAPs among nurses.

**Methods:**

A descriptive cross-sectional design was conducted using a convenience sample of 307 primary health care nurses in Jordan. A self-reported questionnaire (KAPs) toward patient safety was distributed to the nurses between August 2022 and October 2022.

**Results:**

The results revealed that the mean score of knowledge was 9.51 out of 11 (SD = 1.35), the mean score of attitudes was 57.66 out of 75 (SD = 9.17), and the mean score of practices was 5.64 out of 8 (SD = 1.72). Where 59% of participants reported good knowledge about patient safety. 61% of participants reported positive attitudes toward patient safety. A significant regression equation was found (R² = 0.073, F= (2.94), *p* = 0 0.003). Age and having information on patient safety during continuing education were significant predictors of the attitude score (*p* ≤ 0.05).

**Conclusion:**

It is necessary to implement patient safety education programs and training.

## Introduction

Delivering high-quality health care is crucial to ensuring that patients receive the best possible treatment [1]. This includes conforming to qualified healthcare standards, adhering to patient safety measures, and delivering essential services that are accessible, affordable, comprehensive, and well-coordinated [2].

Patient safety (PS), is a major dimension of quality of care and is defined as the avoidance of health care-related errors and adverse outcomes for patients [3]. The Institute for Health care Improvement (IHI) released a report in 2016 which showed that the third leading cause of death in the United States (US) medical errors are one threat to patient safety [4]. In developing countries, the potential of adverse events is far above that in developed states. For example, in the Eastern Mediterranean Region including Jordan, the annual numbers of adverse events are around 4.4 million, and 18% of inpatient admissions were associated with adverse events, with an associated high rate of death and lifelong disability [5]. Recently, the research concerning MEs in Jordan is expanding. However, no previous comprehensive review of literature regarding MEs mortalities in Jordan has been undertaken.

Primary healthcare (P.H.C) is a fundamental approach to healthcare that addresses people’s needs across the continuum of health promotion, disease prevention, treatment, rehabilitation, and palliative care [1]. Primary healthcare centers (P.H.C. Cs) are often the initial point of contact for individuals and provide care to a significant portion of the population, and play a vital role in ensuring PS as the first line of encounter [6]. Furthermore, Primary health care plays a crucial role in preventing the need for higher levels of care and reducing patient vulnerability and exposure to risk factors such as cognitive-, environmental-, social-, and organizational-level factors [7, 8].

While hospitals handle more serious illnesses requiring specialized care, the value of P.H.C services is sometimes underappreciated. Inadequate infrastructure, lack of procedures, and safety standards in P.H.C centers can lead to preventable morbidity and mortality, as well as overuse of hospital resources [7]. Despite being considered lower risk, errors and adverse events can occur in P.H.C centers, and research on PS in these settings is limited compared to hospitals [8, 9]. While PS has traditionally been focused on hospitals, primary healthcare settings play a vital role as the first point of contact for patients. However, PS in primary healthcare is sometimes neglected, resulting in a lack of research and initiatives to improve safety in these settings [10].

To improve PS in primary healthcare, health care facility administrators must prioritize the establishment of a safety culture. This involves fostering a proactive approach to risk mitigation and ensuring that organizational culture supports PS initiatives. A culture of safety should be multidisciplinary and involve the commitment and participation of all healthcare professionals [11]. The collaborative practice among healthcare providers is also vital, as obstacles to interprofessional collaboration can lead to substandard care and unsafe practices.

Nurses play a crucial role in enhancing PS in primary healthcare settings due to their direct involvement in patient care and their ability to detect risks and advocate for patients [12]. Therefore, nurses should be equipped with the necessary knowledge and attitudes towards improving the quality of care and safe practices.

Raising nurses’ awareness and knowledge of PS culture can be achieved through regular risk assessment surveys in primary health centers and the assessment of PS culture among healthcare practitioners [12, 13]. Additionally, PS training should be integrated into the General Nursing Orientation (GNO) when nurses are employed in primary health centers. Moreover, improving nurses’ communication skills and providing training on stress recognition and event reporting can encourage them to identify and address weaknesses in PS practices.

In Jordan, the primary healthcare sector is an essential component of the healthcare system, providing basic health services to the population including health promotion, disease prevention, maternal and child health, and chronic disease management. The government has taken various measures to improve PS in primary healthcare facilities, including the implementation of the Medical Liability Act and the establishment of the National Council for Accreditation of Health Institutions. The government has also collaborated with international organizations, such as the WHO, to strengthen the capacity of health care workers in primary health centers to provide safe and quality care to patients [14]. Despite the importance of PS, studies examining nurses’ perceptions and practices in this regard in Jordan and the region are rare.

It is important to differentiate between primary health centers (P.H. Cs) and comprehensive health care centers (C.H.C. Cs), as Primary centers focus on basic health services and preventive care, while Comprehensive centers offer a more comprehensive range of services, including secondary care and specialized services beside primary services [15]. So, PS is a critical aspect of healthcare quality, and it is imperative to establish a culture of safety in primary healthcare settings. While hospitals have traditionally received more attention in PS initiatives and research, primary healthcare plays a crucial role as the first point of contact for patients. Nurses, as frontline healthcare providers, have a significant responsibility in enhancing PS in primary healthcare facilities. However, a review of the current literature in the Jordan has revealed that no research is available about this subject. The aim of study to assess Knowledge, attitudes, and practices (KAPs) toward PS among nurses working at primary and comprehensive health care centers.

## Methodology

### Study design

A descriptive cross-sectional design was used.

### Study settings

The study focused on primary and comprehensive health care centers affiliated with the Ministry of Health (MOH) in three governorates of Jordan: Amman, Irbid, and Karak. Amman, the capital city, is located in the central part of Jordan and has a population of 4,744,700. It comprises 88 primary and comprehensive health centers, which collectively received 3,478,605 patient visits in 2021. Irbid, situated in the north, has a population of 2,095,700 and encompasses 99 primary and comprehensive health centers, receiving 1,273,468 patient visits in 2021. Karak, located in the south, has a population of 374,800 and comprises 43 primary and comprehensive health centers, with 466,626 patient visits in 2021 [14]. Each region was represented in the health centers’ sample by selecting the number of health centers according to the total number of health centers. The selection of health centers from each region was made according to the percentage of each type in each region. Health centers with a capacity of fewer than 5 beds were excluded because of the small number of registered nurses on their duty schedules.

For this study, a total of 7 primary centers and 8 comprehensive centers were recruited in Amman. In Irbid, 16 primary health care centers and 3 comprehensive health care centers were included. Similarly, in Karak, 13 primary health care centers and 4 comprehensive health care centers were selected. The selection of these health centers was done conveniently to ensure an adequate sample size for the study.

### Study sample

The target population consisted of nurses working in these settings, specifically in the governorates of Amman, Irbid, and Karak. A convenience sample of 307 nurses was selected from the accessible population, which included nurses affiliated with the MOH in the selected governorates. Eligible participants were informed about the purpose and significance of the study, as well as the confidentiality and voluntary nature of their participation. Nurses were included if they had at least a Bachelor’s degree in nursing with at least one year of nursing experience in health centers. Nurses working in hospitals areas were excluded.

### Study tool and data collection

The study employed a validated questionnaire that was adapted from a previous study on nurses’ knowledge, attitudes, and practices related to PS [16]. The questionnaire has been submitted for rigorous review by experts and was translated into Arabic to ensure its suitability and consistency. It comprised sections covering socio-demographic data, personal-related characteristics, knowledge, attitudes, and practices. The nurses’ knowledge section contained 11 items, all of which were scored on a two-level scale of Yes or No, in which Yes answer was coded as 1 and No answer as zero (0). The attitudes of nurses were measured using a Likert scale with five levels on 15 items (Strongly Disagree, Disagree, Neutral, Agree, and Strongly Agree). Where strongly agree was coded as 5, agree as 4, neutral as 3, disagree as 2, and strongly disagree as 1. The nurses’ practices section included eight items, each of which was scored on a two-level scale of Yes or No, in which Yes answer was coded as 1 and No answer as zero (0). Test–retest reliability was done using Kappa ≥ 0.4. The Cronbach’s alpha for the scale in the current study was 0.87.

The data collection process took place between August and October 2022, during which the researchers distributed the questionnaires and provided assistance to the participants as needed.

### Data analysis

The collected data were analyzed using version 26 of Statistical Package for the Social Sciences (SPSS) software. Descriptive statistics were used to summarize the demographic characteristics of the participants, as well as their knowledge, attitudes, and practices regarding PS. Multiple regression models were employed to explore the predictors of these variables. The study classified responses as good or poor based on the mean scores. Nurses who scored equal to or above the mean were considered to have good knowledge, positive attitudes, or good practices regarding PS. Conversely, those who scored below the mean were categorized as having poor knowledge, negative attitudes, or poor practices in relation to PS [16].

## Results

### The socio-demographic characteristics of the study sample

In this study, 307 nurses from three governorates in Jordan completed the questionnaires, resulting in a response rate of 87.7%. The majority of participants were female (88.9%), with a mean age of 37.9 ± 6.48 years and an average of 15.2 years of experience in their current job. Most participants were married (83.7%), 51.1% had a diploma, and 35.5% had a bachelor’s degree. The nurses were recruited from primary centers (60.6%) and comprehensive centers (39.4%) (Table 1).

**Table 1:** The Socio-Demographic Characteristics of the Study Sample (*N* = 307)

Gender:		
Male	34 (11.1%)	
Female	273 (88.9%)	
Age		37.9 (6.48)
Experience		15.2 (6.98)
Weekly job hours		37.5 (5.24)
City:		
Amman	136 (44.3%)	
Irbid	88 (28.7%)	
Karak	83 (27%)	
Marital status:		
Single	39 (12.7%)	
Married	257 (83.7%)	
Divorced	8 (2.6%)	
Widowed	3 (1%)	
Qualifications:		
High school	30 (9.8%)	
Diploma	157 (51.1%)	
Bachelor degree	109 (35.5%)	
Postgraduate	11 (3.6%)	
Center:		
Primary	186 (60.6)	
Comprehensive	121 (39.4)	
Job description:		
*Aid nurse	50 (16.3%)	
**Practical nurse	93 (30.3%)	
***Registered nurse	111 (36.2%)	
Head nurse	13 (4.2%)	
Midwife	40 (13%)	
Department:		
ER	60 (19.5%)	
Dental care	40 (13%)	
Vaccination	8 (2.6%)	
Administration	6 (2%)	
Maternity	51 (16.6%)	
All sections	142 (46.3%)	
Having another job:		
Yes	106 (34.5%)	
No	201 (65.5%)	

*Aid nurse: pre-diploma or high school graduate nurse

**Practical nurse: diploma graduated nurse

***Registered nurse: bachelor’s degree graduated nurse

### Knowledge, attitudes, and practices among study participants

The findings revealed that the mean knowledge score was 9.51 out of 11, (SD = 1.35), based on that the knowledge were divided into two groups (those with poor knowledge (41%) < 9.51 and those with good knowledge (59%) ≥ 9.51). The mean attitude score was 57.66 out of 75, (SD = 9.17), based on that, 61% of participants had a positive attitude toward PS and 39% of participants had negative attitudes toward PS. The mean practice score was 5.64 out of 8, (SD = 1.72), based on that, 61% of participants had a good practice of PS and 39% of participants had a poor practice of PS (Table 2).

**Table 2:** Knowledge, attitudes, and practices among study participants (*N* = 307)

	N	(%)
Knowledge		
Good Knowledge	181	59%
Poor Knowledge	126	41%
Attitudes		
Positive Attitudes	187	61%
Negative Attitudes	120	39%
Practice		
Good Practice	188	61%
Poor Practice	119	39%

### Knowledge of patient safety among study participants

Regarding knowledge about PS, most of the participants recognized the clinical environment as a potential cause of errors (89.6%). However, only 50.2% believed that medical errors are a sign of incompetence. The majority (82.4%) acknowledged the importance of the patient’s role in preventing errors, and 93.2% recognized culture as a key dimension of PS. Additionally, 85% of participants understood that a mistake is a failure to execute an action plan as intended or the implementation of the wrong plan. Almost all participants (96.7%) acknowledged the existence of contributing factors to clinical error occurrence. (Table 3).

**Table 3:** Knowledge of Patient Safety among Study Participants (*N* = 307)

1	The clinical environment can be a cause of errors	
	No	32 (10.4%)
	Yes	275 (89.6%)
2	Medical errors are a sign of incompetence	
	No	153 (49.8%)
	Yes	154 (50.2%)
3	The key to patient safety strategies is set by national health surveillance	
	No	40 (13%)
	Yes	267 (87%)
4	Patients have an important role in preventing errors	
	No	54 (17.6%)
	Yes	253 (82.4%)
5	Human error is inevitable	
	No	76 (24.8%)
	Yes	231 (75.2%)
6	An adverse event is an event that affected the patient	
	No	14 (4.6%)
	Yes	293 (95.4%)
7	Patient safety is the characteristic of a highly reliable health care Organization	
	No	4 (1.3%)
	Yes	303 (98.7%)
8	The key dimension of patient safety is culture	
	No	21 (6.8%)
	Yes	286 (93.2%)
9	A mistake is a failure to execute an action plan as intended or the implementation of the wrong plan	
	No	46 (15%)
	Yes	261 (85%)
10	There are contributing factors to the occurrence of clinical errors	
	No	10 (3.3%)
	Yes	297 (96.7%)
11	There should be a next step to be done after the occurrence of an error	
	No	8 (2.6%)
	Yes	299 (97.4%)

### Attitudes toward patient safety among study participants

Concerning attitudes towards PS, most of the participants agreed that nurse input well received in the clinical area (76.9%) and that it is easy to speak up if they perceive a problem with patient care (75.9%). However, only 63.8% reported that disagreements are appropriately resolved in their clinical area. Most participants agreed that they feel safe being treated in the centers where they work (76.9%) and that medical errors are handled appropriately in their clinical area (69.7%). However, only 61.3% agreed that it is easy to discuss errors in their clinical areas. (Table 4)

**Table 4:** Attitudes toward Patient Safety among Study Participants (*N* = 307)

No.	Items	Strongly disagree N (%)	Disagree N (%)	Neutral N (%)	Agree N (%)	Strongly agree N (%)	Mean (SD)
1	Nurse input is well-received in this clinical area	4 (1.3%)	28 (9.1%)	39 (12.7%)	166 (54.1%)	70 (22.8%)	3.88 (0.90)
2	In this clinical area, it is easy to speak up if I perceive a problem with patient care	1 (0.3%)	26 (8.5%)	47 (15.3%)	175 (57%)	58 (18.9%)	3.86 (0.83)
3	Disagreements in this clinical area are resolved appropriately	11 (3.6%)	33 (10.7%)	67 (21.8%)	149 (48.5%)	47 (15.3%)	3.61 (0.98)
4	I have the support I need from other personnel to care for patients	5 (1.6%)	26 (8.5%)	51 (16.6%)	168 (54.7%)	57 (18.6%)	3.8 (0.89)
5	It is easy for personnel here to ask questions when there is something that they do not understand	5 (1.6%)	16 (5.2%)	39 (12.7%)	180 (58.6%)	67 (21.8%)	3.94 (0.83)
6	The health care workers here work together as a well-coordinated team	6 (2%)	27 (8.8%)	45 (14.7)	161 (52.4%)	68 (22.1%)	3.84 (0.93)
7	I would feel safe being treated here as a patient	7 (2.3%)	12 (3.9%)	52 (16.9%)	163 (53.1%)	73 (23.8%)	3.92 (0.87)
8	Medical errors are handled appropriately in this clinical Area	4 (1.3%)	20 (6.5%)	69 (22.5%)	150 (48.9%)	64 (20.8%)	3.81 (0.88)
9	I receive appropriate feedback about my performance	1 (0.3%)	30 (9.8%)	46 (15%)	156 (50.8%)	74 (24.1%)	3.89 (0.89)
10	I know the proper channels to direct questions regarding patient safety	6 (2%)	26 (8.5%)	31 (10.1%)	191 (62.2%)	53 (17.3%)	3.84 (0.87)
11	In this clinical area, it is easy to discuss errors	6 (2%)	39 (12.7%)	74 (24.1%)	147 (47.9%)	41 (13.4%)	3.58 (0.94)
12	I am encouraged by my colleagues to report any patient safety concerns	5 (1.6%)	25 (8.1%)	64 (20.8%)	161 (52.4%)	52 (16.9%)	3.75 (0.89)
13	This clinical area makes it easy to learn from the errors of others	5 (1.6%)	18 (5.9%)	41 (13.4%)	194 (63.2%)	49 (16%)	3.86 (0.81)
14	Management does not knowingly compromise patient safety	4 (1.3%)	18 (5.9%)	31 (10%)	186 (60.6%)	68 (22.1%)	3.96 (0.82)
15	Fatigue impairs my performance during emergencies	5 (1.6%)	22 (7.2%)	17 (5.5%)	151 (49.2%)	112 (36.5%)	4.12 (0.92)

### Practices toward patient safety among study participants

Regarding practices related to PS, most of the participants reported the presence of teamwork within their unit (84%) and stated that their supervisor’s expectations and actions promote PS were (81.1%). However, only 70.7% reported that there is feedback and communication among staff about errors. While most participants reported a punishment response from the administration for errors (77.2%), the majority also indicated that the centers’ management supports PS (90.6%). Finally, 66.4% reported the presence of organizational learning and continuous improvement in their units. (Table 5).

**Table 5:** Practices toward Patient Safety among Study Participants (*N* = 307)

No.	Items	N (%)
1	Is there Teamwork within the unit?	
	No	49 (16%)
	Yes	258 (84%)
2	Is that Teamwork across the unit?	
	No	120 (39.1%)
	Yes	187 (60.9%)
3	Is that supervisor’s expectation & action promoting patient safety?	
	No	58 (18.9%)
	Yes	249 (81.1%)
4	Are there Feedback and communications about errors?	
	No	90 (29.3%)
	Yes	217 (70.7%)
5	Is there a non-punitive response to error?	
	No	237 (77.2%)
	Yes	70 (22.8%)
6	Does the center management support patient safety?	
	No	29 (9.4%)
	Yes	278 (90.6%)
7	Do the center handoffs and transfer patients?	
	No	38 (12.4%)
	Yes	269 (87.6%)
8	Is there an Organizational learning/continuous improvement?	
	No	103 (33.6%)
	Yes	204 (66.4%)

### Multiple linear regression analysis for the effect of personal and demographic variables on attitude score

The study found no significant predictors for knowledge scores based on participants’ demographics. However, significant predictors for attitude scores included age and having information about PS during continuing education. Older nurses had more positive attitudes, and those who received information about PS during continuing education had more positive attitudes as well. Having information about PS during continuing education was also a significant predictor for good practice scores. (Table 6).

**Table 6:** Multiple linear regression analysis for the effect of personal and demographic variables on attitude score

Predictors	Unstandardized Coefficients		Standardized Coefficients	t	Sig.	95.0% Confidence Interval for B	
	B	Std. Error	Beta			Lower Bound	Upper Bound
(Constant)	41.400	5.254		7.880	0.000	31.060	51.739
Participant Age	0.319	0.121	0.226	2.646	0.009	0.082	0.557
Participant Gender	2.089	1.665	0.072	1.254	0.211	-1.188	5.366
Having information about patient safety during initial education	0.225	1.406	0.012	0.160	0.873	-2.542	2.993
Having information about patient safety during continuing education	3.784	1.541	0.200	2.456	0.015	0.752	6.815
Having training about patient safety	-1.395	1.174	− 0.076	-1.188	0.236	-3.706	0.915
Qualification	0.092	1.142	0.005	0.080	0.936	-2.156	2.339
Experience (years)	-1.332	1.082	− 0.103	-1.230	0.220	-3.462	0.799
Job Description	− 0.638	0.468	− 0.083	-1.363	0.174	-1.559	0.283

Dependent Variable: Total attitude score

*R*² = 0.073

### Multiple linear regression analysis for the effect of personal and demographic variables on practice score

There was a positive significant correlation between the total knowledge score and the total attitude score (*r* = 0.210, *p* = 0.000). Moreover, there was a positive significant correlation between the total knowledge score and the total practice score (*r* = 0.301, *p* = 0.000) (Table 7).

**Table 7:** Multiple linear regression analysis for the effect of personal and demographic variables on practice score

Predictors	Unstandardized Coefficients		Standardized Coefficients	t	Sig.	95.0% Confidence Interval for B	
	B	Std. Error	Beta			Lower Bound	Upper Bound
(Constant)	3.399	0.999		3.403	0.001	1.433	5.365
Participant Age	0.025	0.023	0.095	1.102	0.272	− 0.020	0.070
Participant Gender	0.592	0.317	0.108	1.870	0.062	− 0.031	1.215
Having information about patient safety during initial education	− 0.149	0.267	− 0.042	− 0.559	0.577	− 0.676	0.377
Having information about patient safety during continuing education	0.706	0.293	0.198	2.409	0.017	0.129	1.282
Having training about patient safety	− 0.247	0.223	− 0.072	-1.106	0.270	− 0.686	0.192
Qualification	− 0.276	0.217	− 0.078	-1.272	0.204	− 0.704	0.151
Experience (years)	− 0.012	0.206	− 0.005	− 0.058	0.954	− 0.417	0.393
Job Description	0.017	0.089	0.012	0.194	0.846	− 0.158	0.192

Dependent Variable: Total practice score

*R*² = 0.05

### Correlations between the knowledge, attitude, and practice scores toward patient safety among study participants

There was a positive significant correlation between the total attitude score and the total practice score (*r* = 0.629, *p* = 0.000) (Table 8).

**Table 8:** Correlations between the Knowledge, attitude, and Practice Scores toward Patient Safety among Study Participants (*N* = 307)

		Total knowledge score	Total attitude score	Total practice score
Total knowledge score	Pearson Correlation	1	0.210^**^	0.301^**^
	Sig. (2-tailed)		0.000	0.000
Total attitude score	Pearson Correlation	0.210^**^	1	0.629^**^
	Sig. (2-tailed)	0.000		0.000
Total practice score	Pearson Correlation	0.301^**^	0.629^**^	1
	Sig. (2-tailed)	0.000	0.000	

** Correlation is significant at the 0.01 level (2-tailed)

## Discussion

### Socio-demographic characteristics

The study findings indicate that the majority of participants were females, which aligns with similar study conducted in Egypt [17]. This may reflect the predominance of females in the nursing profession, particularly in primary healthcare and maternal and childcare units. Additionally, the average length of experience among participants was approximately 15 years, suggesting that nurses in primary centers tend rarely to transfer to other settings, as opposed to hospital settings. The study also revealed that around half of the participants held a diploma degree, which may be attributed to the employment policies of the MOH. Nurses with diploma degrees are typically hired in primary healthcare centers, while those with bachelor’s degrees are more likely to be employed in hospitals. Finally, approximately one-third of the nurses reported having another job alongside their primary center employment, potentially driven by the need to improve their financial situation in challenging economic conditions and the flexibility of morning shifts allowing for additional work in the afternoons.

### Knowledge toward patient safety among nurses

The study findings indicate that approximately 65% of the participants had received PS knowledge during their early education, which is lower than similar studies conducted in Ethiopia16, which showed that 73.8% of nurses had knowledge of PS during their early education. On the other hand, the current study result is considered higher than another study that was also conducted in Ethiopia and revealed that 46% of nurses had knowledge about PS in their early education [18]. These differences could be attributed to variations in samples and settings. However, newly employed nurses in primary centers need to have PS knowledge, as they have direct contact with patients and are responsible for their safety. The employment of nurses without early PS training suggests a failure on the part of healthcare centers to provide such training. Furthermore, over 60% of participants acquired PS knowledge during continuing education, aligning with a study in Ethiopia [16]. Additionally, 52.4% of the participants reported having received specific PS training, which is higher than previous studies in Ethiopia. The variance in percentages could be due to the irregular or non-continuous nature of specific training provided either within or outside the healthcare centers in those studies.

The study found that 59% of the participants had good knowledge about PS. This percentage is similar to a study conducted in Ethiopia 16, but lower than a study in Brazil [19]. However, it is higher than another study conducted in Ethiopia [18], which can be attributed to variations in sample size, socioeconomic characteristics, and the number of nurses who received PS training. In terms of specific knowledge about PS, the current study showed that a higher percentage of participants recognized the clinical environment as a cause of errors and understood medical errors as a sign of incompetence compared to the Ethiopian study [18]. Additionally, a majority of participants in the current study identified culture as the key dimension of PS, indicating a higher overall knowledge level compared to the Ethiopian study [18]. Curiously, no significant associations were found between knowledge level and participant demographics in the current study, suggesting that the majority of participants in this study reported good PS knowledge regardless of their demographic characteristics, and the knowledge is gained through experience and continuous education regardless of their qualification degree. This differs from a study in Egypt 20 and a study in Ethiopia 18 that observed significant relationships between knowledge scores and age, qualification, work experience, and training.

### Attitude toward patient safety among nurses

In the current study, 61% of the participants demonstrated a positive attitude towards PS. This percentage was higher than a study conducted in Ethiopia 16 but lower than a study conducted in Egypt [20]. The variations in percentages could be attributed to different settings and the implementation of positive initiatives and educational programs in certain healthcare centers more than our study health centers. Regarding speaking up about PS concerns, only 25% of participants found it challenging to express their opinions and show concern. This percentage was lower than a Jordanian study that was carried out in 91 accredited P.H.C.CS which showed that 39% of the nurses had difficulty speaking up about PS concerns in their centers 17, but it is considered higher than a study conducted in Kuwait [21]. The fear of punishment and negative consequences might explain the difficulty in speaking up reported by nurses in this study.

Most of the participants (76.9%) felt safe being treated as a patient in their centers, which was consistent with a similar Jordanian study [17]. The study also revealed that the majority of participants (79.5%) knew the proper channels to direct questions regarding PS. However, a significant portion found it difficult to discuss medical errors in their areas. This reluctance might be due to concerns about reporting consequences. Additionally, over 70% of the sample reported accessibility to learning from the errors of others, indicating the presence of effective leadership. Age and acquiring knowledge through continuing education were identified as predictors of positive attitude scores, aligning with previous studies that emphasized the importance of ongoing education in shaping positive attitudes towards PS [22–24].

### Practice toward patient safety among nurses

The study demonstrated that over 60% of participants exhibited good practice in PS, which can be attributed to their positive levels of knowledge (59%) and attitudes (61%). In comparison, a study in Ethiopia reported a lower percentage of nurses with good practice (50%), possibly due to the absence of previous improvement initiatives [16]. Teamwork levels across primary centers were reported at 60.9%, lower than a study in Kuwait (87.8%) [21], indicating the need for more clarified policies and communication tools. Only 22.8% of participants reported a non-punitive response to errors, lower than studies in Tunisia (36.5%) [9], Saudi Arabia (30.7%) [25], and Tehran, Iran (60.4%) [26], suggesting a lack of blame-free culture. However, over 90% of participants reported leadership support for PS, higher than previous studies in Iran (36%) [26] and Kuwait (53.8%) [25]. The only significant predictor of practice was having information about PS during continuing education. Overall, the study highlighted positive practice levels, emphasizing the importance of knowledge, attitudes, teamwork improvement, non-punitive response, and leadership support in enhancing PS.

### Implications

The findings of this study could help administrators and policymakers to detect the need for training sessions, protocols and/or inclusive PS policies to protect patients from any harm that may occur while receiving health care services. in addition, it could help administrators to establish a supportive safety culture by enhancing safety practices and incident reporting among nursing staff in primary centers.

This study can hopefully inspire trainers to create updated training programs for primary health care nurses to help them assess patients. This highlights the need for continuing health education which will eventually enable patients to be disease-oriented, and consequently able to prevent harm resulting from health illiteracy, misinformation, or any incident either during care or at home. Receiving regular and continuous training on PS will make nurses updated, knowledgeable, and outstanding in their careers, which provides them with a feeling of confidence due to having updated knowledge and helping patients obtain the care they need.

This study helps academic personnel by drawing their attention to the necessity of well-informed nursing students who can advocate for their patients in the future. As they become properly educated and prepared to care for their patients and save them from preventable errors that may delay recovery or pose a threat to the patients’ lives. This goal can be achieved by nourishing the curricula of nursing colleges in the area of safety.

The current study could help researchers identify gaps in the PS system from a primary health nurse’s point of view. This will hopefully motivate researchers to conduct further studies based on the findings of the current study which will hopefully provide reliable, valid, and evidence-based data.

### Strengths and limitations

Most of the studies on PS were conducted in acute care settings and hospitals. What is special about this study is that it addressed PS issues in primary and C.H.C. Cs in Jordan, a field which only a few studies choose to address. particularly the area of PS as perceived and practiced by Jordanian nurses. In addition, this study covered rural and urban areas in three governorates which makes it more comprehensive than area-restricted studies.

On the other hand, the study has its limitations which include non-probability sampling which may restrict the generalization of the result findings. Secondly, the questionnaire items are self-reported and might be seen as biased, thus affecting the study finding’s reliability. Moreover, because of the cross-sectional design of the study’s survey, it is difficult to determine the causal relationship between variables.

### Recommendations

The study recommends future researchers use another tool for assessing the KAPs of nurses towards PS (i.e., use more specified and skill-related item questionnaires). Random selection of health care centers is another recommended sampling method. Also, other areas of quality of care such as the effectiveness of care rendered to patients from the perspective of nurses, and patient-centered care are recommended to be studied in future research.

Finally, other designs such as qualitative design are recommended to be used to obtain a more in-depth point of view about PS issues from the practitioners as well as the administrators. Experimental designs that use educational intervention about PS are recommended to be used as well to examine the effect of such intervention on the KAP of nurses.

## Conclusion

PS is one of the crucial dimensions of health care services. Therefore, it has to be considered when these services are rendered to patients. PS is usually perceived as a concept, but it should be seen and practiced as a skill and a positive attitude. PS is assessed mostly in acute care settings and hospitals, but in this study, various PS variables at the primary and C.H.C.Cs were studied. The current study has shown that nurses in health care centers have an acceptable level of PS knowledge, attitude, and practices as compared to low-income countries. However, some PS culture components need more improvement and awareness by primary health care community nurses. This culture must be reflected in nurses’ KAPs, and therefore patients will be safe and protected from harm during receiving the care. There is still a need to enhance these variables at health care centers which are considered the front line of patient and health care, especially through continuous education.

## Data Availability

The datasets used and/or analysed during the current study available from the corresponding author on reasonable request.
